# Wilms tumour treatment in Sudan: A 10‐year single‐centre experience

**DOI:** 10.1002/cnr2.1604

**Published:** 2022-02-09

**Authors:** Mohammed Abdalla, Somaya Hameed

**Affiliations:** ^1^ Faculty of Medicine, Pediatric Department Khartoum Oncology Hospital (KOH), University of Khartoum Khartoum Sudan; ^2^ Faculty of Medicine, Pathology Department Omdurman Islamic University Omdurman Sudan

**Keywords:** status, Sudan, Wilms

## Abstract

**Background:**

In Sudan, the survival of Wilms tumour was reported as 11% in a 2008 study. The impact of establishing paediatric oncology service on survival is studied, and the obstacles of treating Wilms tumour patients were identified.

**Aim:**

This study investigates Wilms tumour treatment outcomes over 10 years at Khartoum Oncology Hospital (KOH).

**Methods:**

All Wilms tumour patients from 2005 to 2014 were analysed retrospectively. Patients received treatment based on the NWTS IV protocol. Patients were analysed for overall survival, and event‐free survival and these outcomes were correlated with age, sex, stage at presentation, and histology.

**Results:**

We analysed 143 files of Wilms tumour patients. The male to female ratio is 1.75. The mean age of patients at diagnosis is 3.5 years. The follow‐up period is 5 years. Most patients (83%) had advanced disease stage 3, and 4. There is a very high abandonment rate 61 (42.6%). The event‐free survival among patients who completed treatment is 75.6%, and is 43.4% for all the (143) patients.

**Conclusions:**

“After initiation of the paediatric oncology service, the survival of Wilms tumour in Sudan is 43%. Abandonment of treatment remains high. Opportunity remains to reduce abandonment and establish a surgical paediatric oncology service to improve outcomes further.”

## 1 INTRODUCTION

There is a shortage of data about the epidemiology, pathology, treatment and prognosis of Wilms tumour from Sudan. Dafalla O. Abuidris et al.^1^ published one study in 2008. They noted a high proportion of locally advanced and metastatic disease (stage III [67.6%] and stage IV [10.8%]) and poor results. This group reported a survival rate of 11%.

Sudan population is 43 746 000.^2^ In 2019, The urban population for Sudan accounted for 34.9%. In 2018, the population aged 0–14 years for Sudan was 40.5%.^3^ A 47% of the population lives below the poverty line. A 26% of people living in Khartoum state is below the poverty line. Poverty increase to 70% in the North Darfur western Sudan.^4^

This study is conducted in the Khartoum oncology hospital (KOH). It is a government hospital and the larger of two oncology centres in Sudan. The hospital is state‐funded. Necessary investigations, chemotherapy and radiotherapy are provided free. The oncology unit treats children from all over Sudan. It is situated in Khartoum, which has a population of 9.0 million. Many patients come from rural areas.

The department of Pediatric haematology‐oncology was established in April 2004. Three full‐time consultants staff the oncology unit. The unit accepts an average of 350 new patients for treatment per year. KOH lacks Intensive care facilities, and there is no paediatric surgery department. The paediatric oncology unit accepts Wilms tumour patients after surgery or biopsy from all over Sudan. General paediatric surgeons from other hospitals perform surgery then refer the patients. A qualified radiation oncologist administers radiotherapy. Due to technical problems, lung and whole abdomen radiotherapy cannot be treated. Three‐D radiotherapy is not available. The radiology department lacks CT and MRI machines.

This study investigates Wilms tumour treatment outcomes over 10 years at KOH.

## 2 METHODS

The study is a retrospective analysis of all patients diagnosed with Wilms tumour in KOH between April 2005 and December 2014. A total of 177 patients are registered as Wilms tumour. A total of 34 patients are excluded from the study because the information is incomplete.

Details of presentation, histology and management were extracted from patient records. All patients were staged radiographically with chest and abdomen CT and during surgery. The national Wilms tumour study group (NWTSG) is used for staging (5). Patients are divided into two main groups. The first group is patients who completed treatment (chemotherapy surgery and radiotherapy), and the rest did not start or did not complete treatment.

Throughout this period, standard practice was that patients were assessed at diagnosis by the paediatric surgeon outside the centre. Primary surgery was undertaken where possible. For tumours considered inoperable, pre‐operative chemotherapy was given after Tru cut biopsy. These tumours were regarded as stage III. The histopathology was reviewed at different labs and by a different pathologist. Chemotherapy and radiotherapy are started as soon as possible. Since surgery and the pathology review were done outside the centre, there was a considerable delay in starting treatment promptly. Chemotherapy was delivered according to NWTSG IV (5)

Relapses were treated with surgery if local only, followed by chemotherapy. Radiation therapy was given for local abdominal recurrence if not given initially. Surgical removal of liver and pulmonary metastases was not available. These patients were treated with chemotherapy only. Radiotherapy for lung and liver metastases is technically not possible. The relapse chemotherapy regimens used include vincristine, actinomycin, doxorubicin, cyclophosphamide, ifosfamide, etoposide and carboplatin.

Outcome at the end of treatment was categorized as (a) alive without evidence of disease, (b) treatment abandonment, (c) death from disease persistent disease (unresectable disease, relapse of disease, or persistent disease after the completion of the full treatment), or (d) death from treatment side effect. Relapsed patients who achieved a complete response after second‐line treatment are considered group (a). Survival time was calculated from diagnosis to the last moment of contact, either by clinic visit or active follow up.

The event was defined as no treatment initiated, incomplete treatment and death. OS and event‐free survival are estimated using the Kaplan–Meier and Log‐rank test by SPSS 24.

## 3 RESULTS

**CHART 1:**
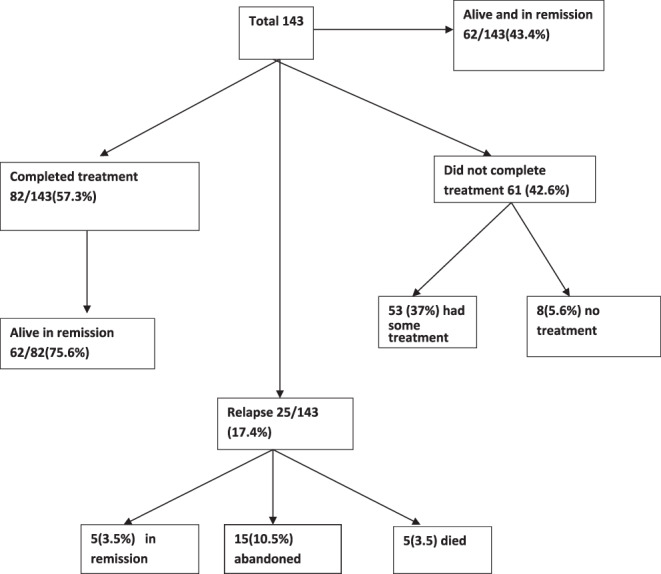
Patients details

Between 2005 and 2014, 2497 children with cancer were registered. A total of 143 (5.7%) patients were confirmed cases of Wilms tumour eligible for evaluation. The male to female ratio is 1.75 (91 male, 52 female). The age range is between 8 months and 12 years, with a mean of 3.5 years. The follow‐up period was up to 61 months. A 100 patients (70%) are between 1 and 5 years (Chart 1).

Chart 1 Pateints distribution

Other characteristics are given in Table 1

**TABLE 1:** Patient's characteristics

Patient's characteristics		
Site	Right	67(47%)
	Left	70 (49%)
	Bilateral	6 (4.2%)
Stage	I	3(2.1%)
	II	16(11.2%)
	III	80(56%)
	IV	39 (27.2%)
	V	5(3.5%)
Histopathology	Favourable	123(86.0%)
	Unfavourable	15(10.5%)
	Undetermined	5(3.5%)
Time of surgery	Upfront nephrectomy	85(59.4%)
	Post chemotherapy	27(18.9%)
	No surgery	31(21.7%)

### 3.1 Surgery

No surgery was performed on 31 patients because they either (a) refused treatment, (b) were lost for follow up, (c) died from chemotherapy side effect, (d) had poor performance status, or (d) had bilateral disease. There was no difference in survival between those children who had primary surgery and those offered neoadjuvent chemotherapy.

### 3.2 Chemotherapy

**FIGURE 1:**
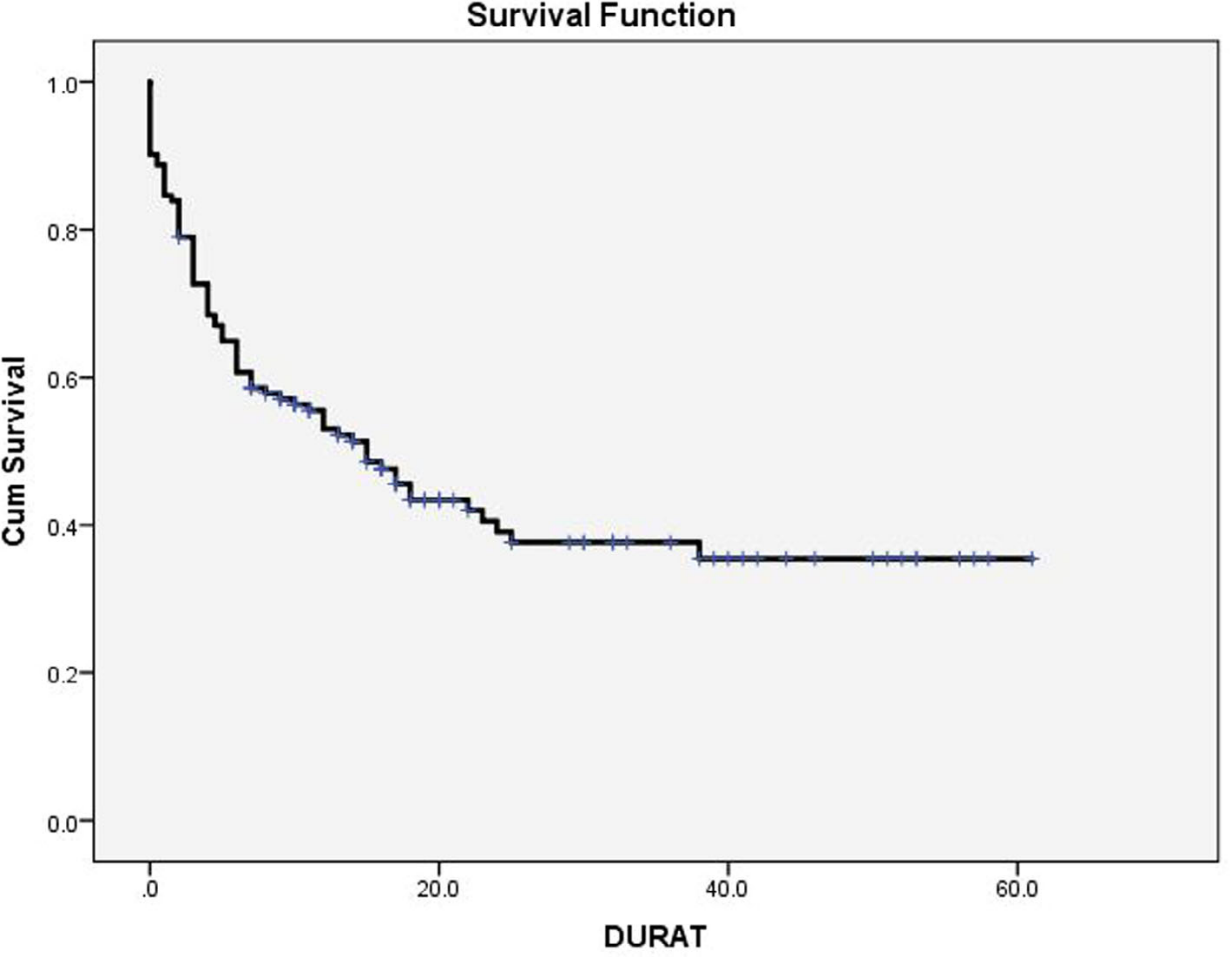
Event free survival. Abandonment and death are considered an event. Event free survival = 43.4%

**FIGURE 2:**
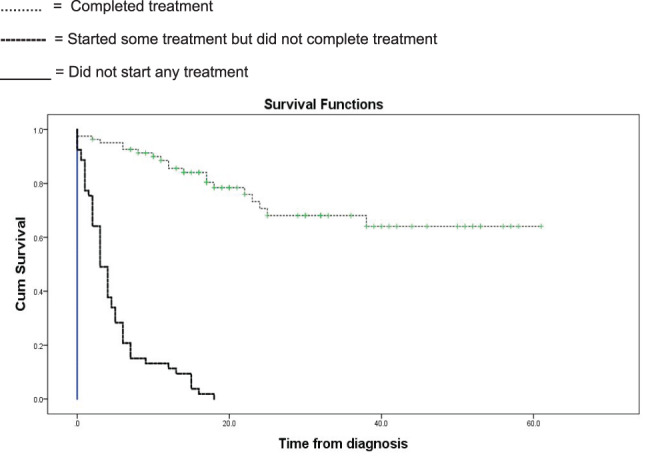
Survival by treatment competition

A total of 82 (57.3%) patients completed therapy (surgery chemotherapy and radiotherapy), while 53 (37%) received some treatment and 8(5.6%) patients did not receive any treatment. The event‐free survival among patients who completed treatment is 75.6%, and the overall survival is 83.4%. The event free survival and OS among the whole 143 patients is 43.4% and 47.6% (Figures 1 and 2). Survival among patients who completed chemotherapy, surgery, and radiotherapy is significantly better than who did not *p* < .01 No chemotherapy was given to 8(6%) patients because they were lost for follow‐up or refused treatment. One patient was given chemotherapy for palliation.

The actuarial survival is 3 (100%) for stage I, 12 (73.3%) for stage II (censored), and 45 (57%) for stage III (censored) 21 (55%) for stage IV and 1916.7%) for stage V.

### 3.3 Abandonment

A total of 61/143 (42.6%) patients abandoned treatment, 32/61 (52%) did so in the first 3 months. Eight patients refused treatment upfront, and one was given treatment as palliation.

### 3.4 Relapses

A total of 25 patients relapsed, constituting 17.4%. The relapse rate is higher among stage III (18) patients and stage IV (6) patients. One patient had progressive disease while on chemotherapy, and three patients had partial/no response. Five patients were salvaged with second‐line treatment and are alive and well when last seen. Five died, and 15 patients were lost for follow up. Most relapses occurred in the patients who completed treatment (19). The most common site of relapse is primary site 17(68%), lung and local 7(28%).).

### 3.5 Deaths

A total of 17 (11.9%) died during the study, 14 were disease‐related, and three were treatment‐related.

## 4 DISCUSSION

This study reflects the situation in the largest oncology hospital in Sudan. Compared to previous studies, the subject number is reasonably large, and follow‐up duration is longer. It is the largest data from Sudan about Wilms' tumour's outcome using the NWTS 4 protocol. It identifies many problems that face Wilms tumour management. The proportion of Wilms tumour to all childhood cancer in our study is (5.7%). The incidence, age and sex distribution are comparable to most international studies and other low‐income countries (LIC) countries.^5^, ^6^, ^7^, ^8^, ^9^ However, these figures are drawn from hospital‐based records and might not reflect the population incidence.

Most of our patients presented with advanced disease stage, stages 3 and 4 constituted (83%) of all cases. This contrasts with other more extensive studies that have the bulk of their patients presenting with early‐stage disease.^10^ It is also higher than other low income and middle‐income countries^5^, ^8^, ^11^, ^12^, ^13^, ^14^ Favourable histology is the most common (86%). It is consistent with a study by Hoda M. Awadalla. In their study, 82.2% of Wilms tumour in Sudanese children was the favourable triphasic type.^15^

Primary nephrectomy is the most frequent type of surgery. All surgeries are performed outside the hospital. There is a significant delay in surgery due to the long waiting lists. There is also a delay in obtaining the histopathology report. Much information is collected through personal communication and imaging. It takes at least 1 month after surgery to start chemotherapy and radiotherapy.

Pre‐operative chemotherapy, followed by nephrectomy appears to be a reasonable approach. There are no surgical facilities at the hospital, making it challenging to adapt the SIOP pre‐operative chemotherapy. Many patients who received neoadjuvant chemotherapy had significant delays when referred for surgery. In some cases, the tumour increased in size significantly. This approach is also challenging to adapt too. Pre‐operative chemotherapy seems the right strategy, but it is impossible to use it now. A project to establish a surgical unit has inside the hospital has started.

Although chemotherapy is free, some families refused to start treatment or discounted therapy early during the disease 61 (43%). This reluctance is due to many financial restrains and social beliefs. Although many patients refused treatment, there was a significant improvement compared to the previous study.^1^ As expected, the survival rate in these two groups is reduced. Our results are comparable to some low‐income countries.^14^, ^16^, ^17^

Because surgery is done outside the hospital, radiotherapy is delayed for more than 1‐month in all cases. This delay might contribute to the poor results.

Age, sex and stage of presentation did not have a statistically significant impact on survival. Treatment completion is the most significant prognostic factor in our study *p <* .0001. It is known that Histopathology type is the most significant prognostic factor.^8^, ^18^, ^19^ Guruprasad et al. found that only the nodal status to be independently associated with survival.

The event‐free 43.3% is comparable to many African and third world countries. Although considered inferior, this is a significant improvement from the previous study in Sudan.^1^ The survival among the patients who completed therapy is reasonable (75%) and comparable to many low‐income countries.^16^ Because most patients (83%) presents with advanced disease, the recurrence rate is high (17.4%).^19^

There is a high local recurrence rate, either isolated 17(68%) or with distant metastasis 6(24%). Most studies report a higher incidence due to lung relapse.^20^ The delay in starting radiotherapy might explain such findings.

We had one of the highest abandonment rates in Africa.^19^, ^20^ The cost of treatment is not the reason; because chemotherapy and radiotherapy are free. Three guesthouses provide free accommodation. The high rate of abandonment is multifactorial. Possible causes include the long‐distance from the facility, poverty, long waiting times and anxiety about what to expect. Illiteracy, many social and religious believes about cancer are other reasons. Close Follow‐up, including home visits when needed, might reduce this problem.^20^ Chagaluka et al.^21^ found that funds used to cover treatment, travel, and other associated costs for patients, significantly reduced the abandonment rate. A different approach to minimize abandonment might be more useful in our setting. A project aiming to give part of the treatment at local hospital will start soon., The death rate is low as compared to Israels et al.^20^and contributes to 11.9% of treatment failure. The department began measures to improve communication with patients with poor compliance.

This study has shown the benefit of a specialized or semi specialized paediatric oncology unit in low‐income countries. The survival rate improved from 11% to overall survival of 43% and 75% for patients who complete therapy. There is a high abandonment rate that needs further attention. Institutionalized measures such as those adopted by L F Chukwuemeka Anyanwu,^16^ might improve results.

The study also highlights some problems that face LIC. It emphasizes the importance of having an in‐house surgical unit to reduce surgical delays and hence radiotherapy delays.

## CONFLICT OF INTEREST

The authors declare no conflicts of interest.

## AUTHOR CONTRIBUTIONS

**Mohammed Abdalla:** Conceptualization (equal); data curation (equal); formal analysis (equal); methodology (equal); project administration (equal); software (equal). **Somaya Hameed:** Conceptualization (supporting); investigation (supporting).

## ETHICS STATEMENT

This study was approved by the Hospital Research committee. Patient approval was obtained verbally, and by phone. Written consent was obtained whenever possible because many lived long distances from the hospital and transportation is very costly. It was not possible to get consent from some patients who abandoned treatment.

## Data Availability

The data sets analysed during the current study are available from the corresponding author on reasonable request.
